# Compassion fatigue, watching patients suffering and emotional display rules among hospice professionals: a daily diary study

**DOI:** 10.1186/s12904-020-0531-5

**Published:** 2020-02-25

**Authors:** Igor Portoghese, Maura Galletta, Philip Larkin, Salvatore Sardo, Marcello Campagna, Gabriele Finco, Ernesto D’Aloja

**Affiliations:** 1 Dipartimento di Scienze Mediche e Sanità Pubblica, Università degli Studi di Cagliari, SS554 bivio per Sestu, 09042 Monserrato, CA Italy; 2 UNIL | Université de Lausanne, CHUV | Centre hospitalier universitaire vaudois, Lausanne, Switzerland

**Keywords:** Patients suffering, Emotional display, Burnout, Secondary traumatic stress, Compassion fatigue, Diary study

## Abstract

**Background:**

Hospice workers are required to regularly use emotional regulation strategies in an attempt to encourage and sustain terminally ill patients and families. Daily emotional regulation in reaction to constantly watching suffering patients may be intensified among those hospice professionals who have high levels of compassion fatigue. The main object of this study was to examine the relationship between daily exposition to seeing patient suffering and daily emotional work, and to assess whether compassion fatigue (secondary traumatic stress and burnout) buffers this relationship.

**Methods:**

We used a diary research design for collecting daily fluctuations in seeing patients suffering and emotional work display. Participants filled in a general survey and daily survey over a period of eight consecutive workdays. A total of 39 hospice professionals from two Italian hospices participated in the study.

**Results:**

Multilevel analyses demonstrated that daily fluctuations in seeing patients suffering was positively related to daily emotional work display after controlling for daily death of patients. Moreover, considering previous levels of compassion fatigue, a buffering effect of high burnout on seeing patients suffering - daily emotional work display relationship was found.

**Conclusions:**

A central finding of our study is that fluctuations in daily witness of patients suffering are positively related to daily use of positive emotional regulations. Further, our results show that burnout buffers this relationship such that hospice professionals with high burnout use more emotional display in days where they recurrently witness patients suffering.

## Background

Over the last decade, access to palliative care and hospice services have grown rapidly around the world [1]. Recently, the World Health Organization emphasized the need for improving the quality of life of patients and relatives facing the problem of life-threatening illness by addressing their physical, psychological, social and spiritual needs [2–4]. In this sense, hospice care professionals (HCPs) provide intensive interventions aimed at improving quality of life and relieving suffering [5, 6].

According to a recent systematic review on wellbeing of HCPs, “there is relatively little research to address the psychological wellbeing of the staff” who deal with death and dying on a daily basis in hospice context (p. 2) [7].

Working in palliative care context may expose staff to recurrent distressing events on a daily basis, such as exposure to death and dying, patient suffering, and observing extreme physical pain in patients, resulting in the risk of absorbing negative emotional responses, coping with inability to cure and potentially, deep engagement in emotional clashes [8–13]. It has been calculated that 50% of HCPs are at risk of reduced psychological well-being as a result of inadequate organizational strategies related with many of these demands [14].

Among those stressors that may affect staff emotional work, limiting HCPs true emotions as health care workers, witnessing the extreme suffering of patients represents an intense challenge for HCPs in terms of emotional management, ethical obligations and personal integrity as individuals and professionals [15–17]. Working in hospice context entails daily recurrent and intense interactions with patients and families that require regular use of emotional labor regulation strategies which may lead to reduced well-being [5, 16, 17]. Emotional labor has been defined as the effort involved when workers “regulate their emotional display in an attempt to meet organizationally-based expectations specific to their roles” (p. 365) [16]. Furthermore, emotional labor is linked to perceived display rules defined as those shared expectations around what emotions workers should and should not show. Specifically, displaying positive emotions (salutogenic factor) and suppressing negative ones (pathogenic factor) are common rules in hospice context, and are considered as in-role (emotional) job requirements [17, 18]. For example, displaying positive emotions during social interactions with patients and families as part of their role as clinicians in attempt to influence (positively) patients’ attitudes and behaviors, encouraging and sustaining patients and family [19].

According to Joinson [20], this intense and recurrent emotional labor may expose HPCs to vicarious stress and development of compassion fatigue (CF). CF is defined as “a state of tension and preoccupation with traumatized patients by re-experiencing the traumatic events, avoidance/numbing of reminders and persistent arousal associated with the patient” [21, 22]. According to the Compassion Stress/Fatigue Model [23, 24], CF has been considered as the resulting caregivers’ behaviors and emotions linked to knowing about a traumatizing event experienced or suffered by a person” [22, 25] and the resulting reduced capacity or interest of those in “bearing the suffering of clients”.

In this sense, HCPs are at high risk of developing CF as they provide prolonged involvement and compassion for those who are suffering, frequently without seeing patients improving [26]. Furthermore, not only prolonged or continuous exposure to stressful events may play a crucial role in generating CF, but a single intense event may also be decisive. Hereafter, CF is the fatigue associated to constantly dispensing compassion, day after day [27].

CF has been theorized as a multi-component construct, comprised of secondary traumatic stress (STS) and burnout [23, 28]. STS has been defined as the condition when care providers report symptoms related to reexperiencing the traumatic experience of patients (vicariously experience) [23]. Burnout is a form of cumulative work related stress and is characterized by emotional exhaustion, cynicism, and reduced personal accomplishment [29]. While CF is considered as a form of reaction to traumatic patient experience, job burnout is associated with workplace context, such as high job demands, low job control, and low job support [30].

Simon, Pryce, Roff, and Klemmack [31] found that working with dying patients exposed workers to secondary traumatic stress and that it was the recurrent emotional demand that led to CF. Hence, HCPs suffering from CF may be not able to effectively regulate their emotional display [32]. In this sense, an additional implication is that being in a condition of CF may booster the effect of witnessing suffering patients on emotional display. Thus, we hypothesized that the effects of witnessing suffering patients on emotional display rules would depend on the HCPs’ previous levels of CF, such that this relationship should be strongest for those HCPs with higher CF.

As most of the stressors are likely to occur within the same workday, the main purpose of the present study was to investigate the relationship between potential short-term fluctuations in witnessing suffering patients and daily use of positive display emotion rules.

## Methods

### Participants and procedure

At the time of this study, there were two not-for-profit hospice organizations in the Local Social Health Area (ASSL). Both hospices were approached by the research team to inform them of our study and both agreed to participate in our study. In the first hospice (18 beds), a staff of 28 hospice professionals provide care for cancer patients. In the second one (12 beds), a staff of 22 hospice professionals provide care mainly for patients with cancer, dementia, multiple sclerosis, ALS, and other serious illness that has received a terminal diagnosis. The target population for the present study included physicians, registered nurses (RNs), psychologists, and health assistants who met the following inclusion criteria: (1) will be still employed by a hospice organization during the study (1 month), and (2) interact directly with patients and their families. A total of 47 healthcare workers were eligible to be involved in the study. Of those, 41 accepted to participate. All participants received written information about the aims of the research and gave their written informed consent. Participation was voluntary, there was no adverse consequence of declining or withdrawing from participation, and confidentiality was protected since responses were kept anonymous. Participants received no incentive for their involvement.

Considering the aims of our study, we adopted a diary research design [33, 34]. With this methodology it is possible to analyze fluctuating workplace experiences by collecting data at the daily\weekly level. When compared to traditional research design (cross-sectional or longitudinal), diary methods offer the opportunity to capture short-term fluctuations of variables within and between individuals [35]. Initially, participants received a general questionnaire aimed to collect socio-demographic information and compassion fatigue. After two weeks they received a package including a diary booklet and instructions on how to complete the daily diary (eight daily diaries, one diary every working day). The participants were also asked to fill in a personal code on the questionnaire and the diary booklet allowing us to match their responses to each questionnaire. Completed questionnaires were returned to the research team in an anonymous closed envelope.

A total of 39 hospice professionals participated in the study, resulting in a response rate of 95.1%. Two hospice professionals completed less than 50% of the diaries and then when removed from the analyses. 44% were nurses, 12% physicians and 44% other healthcare professionals (psychologists and health assistant). Overall, 76% of respondents had been working in their respective hospice for between 4 and 10 years.

### Measures

#### Questionnaire data

We assessed socio-demographic information as well as compassion fatigue through a general questionnaire that had to be completed once, before the diary surveys. As requested from the workers, to ensure anonymous response, we did not include sex and age in the questionnaire.

Compassion fatigue was measured using the Professional Quality of Life Assessment R-IV Scale (ProQOL-RIV) [30]. Specifically, in the present study the burnout (10 items) and Secondary Traumatic Stress Scale (STSS 10 items) were used. Response options ranged from 0 = never to 5 = always. Cronbach’s alpha for burnout and STSS were respectively 0.74 and .87.

#### Diary data

The diary booklet assessed daily fluctuations of two emotional job demands (death of a patient and watching a patient suffer), and emotional work requirements (displaying positive emotions).

##### Daily emotional job demands

Two items from the Nursing Stress Scale [36] were adapted to measure the frequency of two specific emotional demands: “the death of a patient with whom you developed a close relationship” and “watching a patient suffering”. Response options ranged from 1 (never today) to 4 (very frequently today).

##### Daily emotion work display

We adapted three items from the Emotion Work Requirements Scale [37] to assess hospice workers emotional display rules. Specifically, displaying positive emotions to patients and relatives. Furthermore, based on a review of the emotional labor literature and regulating emotions by displaying feelings as part of the work role in hospice context, we added three items: “I had to put one or more patients in a good mood”, “I easily expressed my positive emotions to the patients” and “I easily expressed my positive emotions to the relatives of the patients”. Response options ranged from 1 (never today) to 4 (very frequently today). We inspected the factor structure of this measure at both the between- and within-person levels using the multilevel confirmatory factor analysis (MCFA). A maximum likelihood estimation procedure was used. We considered (a) the Comparative Fit Index (CFI) [38], with values > 0.90 suggesting an adequate fit; (b) the Standardized Root Mean Square Residual (SRMR) [39], with values < 0.08 suggesting acceptable fit, and the (c) Root Mean Square Error of Approximation (RMSEA) [40], with values < 0.08 suggesting acceptable fit. The MCFA confirmed a one factor solution at both within and between-level. This model yielded acceptable fit: χ2 = 48.69; df = 17; *p*-value < 0.001; CFI = 0.91, and the SRMR between = 0.262 and SRMR within = 0.047; the RMSEA = 0.077.

Cronbach’s alpha ranged from 0.72 to 0.92 over the eight diaries (mean α = 0.81).

### Analytical strategy

As our data are a two-level hierarchical structure, repeated measurements (days) nested within individuals, we inspected our model using hierarchical linear modeling (HLM Version 6) [41]. We estimated the fixed and random parameters by usingd the restricted maximum-likelihood procedure in HLM. We centerd the Level 2 data on the grand mean and Level 1 on the respective person mean. In order to test whether HLM analyses were appropriate, within-person and between-person variance components were investigated [34, 42].

## Results

### Preliminary analyses

Firstly, we examined the between-persons and within-person variance components of the variables. Specifically, we inspected reliability of the estimates of the level 1 intercepts and intraclass correlation (ICC) by running null models with no predictors (besides the intercept).

Results showed that between-person variation accounted for 33.26% of the variance in daily emotion work display, 38.1% of the variance in daily witnessing a patient suffering, and 7.6% of the variance in daily death of a patient with whom they developed a close relationship. ICCs of daily emotion work display and daily watching a patient suffer were above the minimum suggested (ICC > 0.10), justifying running HLM analyses. The variable daily death of a patient with whom they developed a close relationship was considered as control variable. All variance components were significant at *p* < 0.01.

Means, standard deviations, and correlations for all the study variables are presented in Table 1. All significant relationships between the variables were in the expected direction.

**Table 1:** Means, standard deviations and correlations within each level of analysis

Level 1	M	SD	1	2	3
1. Number of dead patients (per day)	1,31	0,65	–		
2. Watching patients suffering (per day)	2,19	0,84	−0,06	–	
3. Daily positive emotion work display	2,53	0,62	−0,01	0,22	–
Level 2	M	SD	**1**	**2**	
1. Burnout	2,04	0,70	–		
2. STS	1,36	0,86	,75**	–	

*Note*. All variables are within-person (Level 1, *n* = 312) variables except the between-person variables burnout and STS (Level 2, *n* = 39);

**p < .01

### Tests of the hypotheses

According to the Hypothesis 1a, watching a patient suffer would be related to emotion work display at the intra-individual level (Table 2). In testing our hypothesis, we started with a null model that included the intercept as the only predictor. Next, in Model 1, we added number of patients who died (γ = .02, *ns*) as control variable at level 1 in HLM and daily watching patients suffering at level 1 in HLM. Results showed that daily watching patients suffering was significantly and positively related to daily positive emotion work display (γ = .19, *p* < .01) supporting the hypothesis 1.

**Table 2:** Multilevel estimates for daily positive emotion work display

	Null			Model 1			Model 2		
	Est	SE	t	Est	SE	T	Est	SE	t
Intercept	2,52	.06	41,31***	2,52	0,06	41,31***	2,53	0,06	41,28***
Level 1									
Daily number of dead patients				0,02	0,04	0,42	0,02	0,04	0,49
Daily watching patients suffering (DWPS)				0,19	0,06	3,19**	0,20	0,06	3,44**
Level 2									
Cross-level moderation 1 (STS*WPS)							−0,05	0,10	−0,53
Cross-level moderation 2 (burnout* DWPS)							0,18	0,08	2,28**
Level 1 Intercept Variance	0,13			0,13			0,13		
Level 2 Intercept Variance	0,26			0,22			0,22		

*Note*. N = 39 employees and N = 312 observations

***p < .001; ** *p* < .01; *p < .05

### Cross-level moderating effects of compassion fatigue

**Fig. 1:**
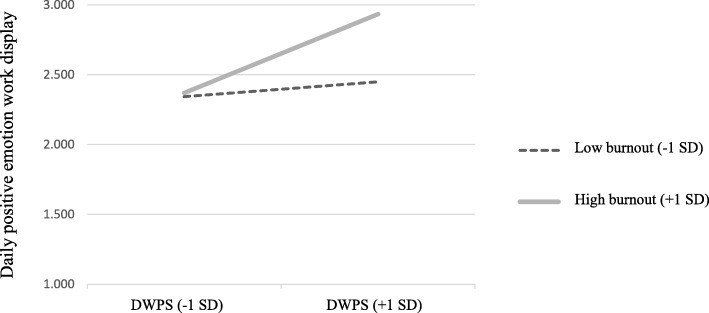
Cross-level interaction. Moderation of burnout in the relationship between watching patients suffering (DWPS) and daily positive emotion work display

Concerning the cross-level moderating effect of compassion fatigue, we analyzed the simultaneous effect of both burnout and STS in the relationship between watching patients suffering and daily positive emotion work display, results (see Table 2, Model 2) revealed that only cross-level moderation effect of burnout was significant (γ = .18, *p* < 0,01). Simple slope tests results showed (Fig. 1) that this relationship was stronger when burnout was high (γ = .34, *p* < .01) than when it was low (γ = .06, ns). Finally, the cross-level moderating effect of STS was not significant (γ = −.05, *ns*).

## Discussion

HCPs work constantly in an emotionally challenging context [43] and are vulnerable to compassion fatigue, burnout and emotional related issues [22, 31, 44, 45]. The main purpose of this study was to examine the relationship between daily fluctuations in seeing patient suffering and daily emotional work display, and to assess whether CF (STS and burnout) moderate this between-person relationship.

At the between-person level, results from our study were in line with previous research that showed how emotionally demanding jobs entail a higher frequency and intensity of daily interactions with patients and families that in turn requires regular use of emotional labour regulation [16, 45]. In this sense, on days where HCPs witness suffering patients frequently, they will regulate their positive emotional display. In this sense, regular use of emotional labour regulation strategies can expose HCPs to reduced well-being [15, 16, 46, 47]. Our results are in line with traditional studies that demonstrate how emotional connections are a vital component of the therapeutic relationship in the hospice context, expressing their feelings when healing suffering is a fundamental part of this relationship [46, 47].

Additionally, in relation to the cross-sectional moderation effect, we found that burnout moderated the within-person relationship between seeing patients suffering, and daily emotion work display such that this relationship was stronger for those high in burnout.

Concerning secondary traumatic stress, we did not find support for the moderation effect.

The existing theory of, and research into, emotional management and compassion fatigue among HPCs has been almost exclusively cross-sectional and at the between-person level of analysis. Using a diary methodology, the current study is one of the first to adopt a time perspective.

### Limitations and suggestions for future research

Despite interesting results, this study has some limitations. First, our sample size as well as the number of daily diaries were modest and that may have reduced statistical power of our results. However, our sample is in line with Scherbaum and Ferreter [48] who suggested sample size (person level) bigger than 30 may to avoid biased results. Second, in line with the diary methodology, we assessed emotional demands using a single-item measure. Single items are very common in diary studies [27] and there is a general agreement that are valid and reliable [49]. In this sense,.Future studies should examine a broader range of emotional demands as we were not able to capture the full range of emotional demands in hospice context. Third, we assessed emotion work by adapting a version of the Emotion Work Requirements Scale. However, we provide acceptable evidence of psychometric properties of this measure.

Finally, as our study is correlational in its nature as all our variables were measured at the same time (although 8 different days). Thus relationships between the studied variables are correlational and conclusions about causality should be made with caution.

#### Practical implications

Findings from our study have practical implications for hospices that strive towards promoting healthy workplaces for their employees. For HCPs regularly confronted with high emotional job demands, emotion regulation strategies are a formal part of their job. Therefore, to reduce negative effects of emotional labor linked suffering patients, it should be crucial for hospice organizations to develop training programs on both emotion recognitionand deep acting strategies. Furthermore, findings from our study also suggest that preventing burnout from becoming too high might actually reduce the impact of seeing patients suffering on emotion work display, too. In this sense, organizations should enhance specific job resources, such as emotional support from colleagues and supervisors for reducing burnout risk.

## Conclusions

In conclusion, this study provides empirical evidence that daily fluctuations in seeing patients suffering are related to HCPs emotional display reactions and that burnout boosts this relationship. Our study should stimulate hospice managers to promote and develop practices to manage emotional demands on a daily basis. A workplace who promotes the development of effective emotional management strategies is beneficial for both HCPs wellbeing and patient’s quality of life.

## Data Availability

Raw data pertaining to analyses performed in this study are available from the corresponding author on reasonable request.

## Abbreviations

CF: Compassion fatigue
DWPS: Daily watching patients suffering
HCPs: Hospice care professionals
HLM: Hierarchical linear modeling
ICC: Intraclass correlation
ProQOL-RIV: Professional Quality of Life Assessment R-IV Scale
STS: Secondary traumatic stress
STSS: Secondary Traumatic Stress Scale
